# Mixed methods evaluation of the inaugural year of the Cancer Prevention and Control Research Network’s (CPCRN) scholars program

**DOI:** 10.1007/s10552-023-01702-1

**Published:** 2023-04-28

**Authors:** Cam Escoffery, Courtney N. Petagna, Mary Wangen, Kimberly J. Flicker, Samuel B. Noblet, Mayank Sakhuja, Cynthia A. Thomson, Elaine H. Morrato, Swann Adams, Jennifer Leeman, Daniela B. Friedman

**Affiliations:** 1 Rollins School of Public Health, Department of Behavioral, Social, and Health Education Sciences, Emory University, 1518 Clifton Road, NE, Atlanta, GA 30322 USA; 2 Center for Health Promotion and Disease Prevention, The University of North Carolina at Chapel Hill, Chapel Hill, NC USA; 3 The University of South Carolina, Arnold School of Public, Health Health Promotion, Education, and Behavior, Columbia, SC USA; 4 Health Promotion Sciences Department, University of Arizona, Mel and Enid Zuckerman, Tucson, Arizona USA; 5 Loyola University Chicago, Parkinson School of Health Sciences and Public Health, Chicago, Illinois USA

**Keywords:** Training, Dissemination and implementation science, Capacity-building, Research network, Cancer disparities, Evaluation

## Abstract

**Purpose:**

A diverse workforce trained in dissemination & implementation (D&I) science is critical for improving cancer outcomes and reducing cancer-related health disparities. This study aims to describe and evaluate impact of the Cancer Prevention and Control Research Network (CPCRN) Scholars Program in preparing scholars for collaborative careers in cancer control and implementation research and practice, and offers evaluation-driven recommendations for program improvements.

**Methods:**

The CPCRN Scholars Workgroup conducted a sequential, mixed methods evaluation. We collected baseline and follow-up surveys and invited all 20 scholars and ten mentors to participate in an exit interview. We assessed the experience with the Scholar’s program, ratings of D&I competences, progress on their project, feedback about the curriculum, and understanding of implementation science.

**Results:**

Over 86% partially or fully completed their project within 9 months; 78% of scholars engaged with a CPCRN workgroup. Scholars rated the following program components as valuable: the Putting Public Health Evidence in Action (PPHEIA) training (88.9%), D&I training modules (83.3%), and webinars (kickoff webinar-88.9% and selecting theories/models-88.9%). There was an increase in D&I competencies from baseline to posttest, with the greatest in community engagement topics. About 78% reported that they were satisfied with format of the activities and increased confidence in ability to discuss D&I concepts. From the qualitative interviews, the benefit of the program was becoming more knowledgeable about D&I research and networking.

**Conclusion:**

The inaugural year of the program yielded positive results, particularly related to increasing knowledge about D&I science and cancer control. This program builds the capacity of students, researchers and practitioners in D&I science.

**Supplementary Information:**

The online version contains supplementary material available at 10.1007/s10552-023-01702-1.

## Introduction

A diverse workforce trained in dissemination & implementation (D&I) science is critical for improving cancer outcomes and reducing cancer-related health disparities. D&I science is the study of methods to promote the systematic uptake of evidence-based interventions and practices to real world context to prevent diseases or reduce their burden [1]**.** Training of students, public health professionals, and researchers is an important strategy to expand this workforce. Over the past two decades, there have been numerous efforts to increase training opportunities in D&I science through workshops, institutes, and degree-seeking programs. The National Institutes of Health’s Training in Dissemination and Implementation Research in Health (TIDIRH) began as a 5-day institute including a balance of structured content topic areas, interactive small-group discussions, and personalized sessions on individual trainee projects [2]. The ultimate goal of the institute was to increase the submission rate and quality of D&I grant applications and scholarly publications among trainees. In later iterations, it has been delivered virtually and an adaptation of it was developed for professionals who worked in cancer control [3]. Additionally, institutions with Clinical Translational Science Awards (CTSAs) have focused on clinical translational science and training; they also have emphasized the importance of D&I science methods to translational science. In one survey, about 70% of CTSA leaders reported they directly or indirectly supported D&I science training; however, they also identified challenges to D&I activities at their institutions, such as funding, limited understanding of D&I science, and a small D&I workforce [4]. Furthermore, schools of public health and other medical institutions have developed degree programs with many at the doctoral or post-doctoral (T32) training in this area [5].

Most existing training opportunities have enrollment limits and many have focused on advancing the knowledge and skills of academic faculty researchers or clinical investigators; thus, there is a D&I training gap for early-stage researchers, such as, graduate students and post-doctoral fellows, and for practitioners employed in healthcare and community-based public health settings. The Cancer Prevention and Control Research Network (CPCRN) was established in 2002 and is a Centers for Disease Control and Prevention (CDC) funded network of eight centers whose mission is to implement evidence-based cancer prevention and control strategies in communities. The CPCRN Scholars program was launched in 2021 to build capacity by nationally scaling available training (e.g., Putting Public Health Evidence in Action-PPHEIA) across all CPCRN sites and beyond, and mentoring students, researchers, and practitioners in D&I science for cancer prevention and control [6]. There are existing D&I competencies that were used to measure scholars’ understanding of D&I science and informed program development. The CPCRN Scholars program uses a combination of teaching modalities: asynchronous digital content for self-paced, self-study; synchronous webinars and small-group discussion; and individualized mentorship on a scholarly project. The program curriculum emphasizes evidence-based public health/cancer interventions and D&I scientific frameworks, strategies, and methods to advance knowledge and increase competencies related to implementation research and practice. Students completed one of two D&I curricula, the Putting Public Health Evidence into Practice Training or NCI’s Training Institute of Dissemination and Implementation Research in Cancer, open access modules [3, 7–9]. They also were invited to learn about the CPCRN and related D&I science through readings, attendance at the annual meeting, network-wide webinars, and workgroup participation [10].

In this paper, we describe the program and the evaluation conducted to test the program’s impact in preparing scholars for collaborative careers in cancer control and D&I science. We also offer recommendations for program improvements guided by evaluation results.

## Methods

**Fig. 1:**
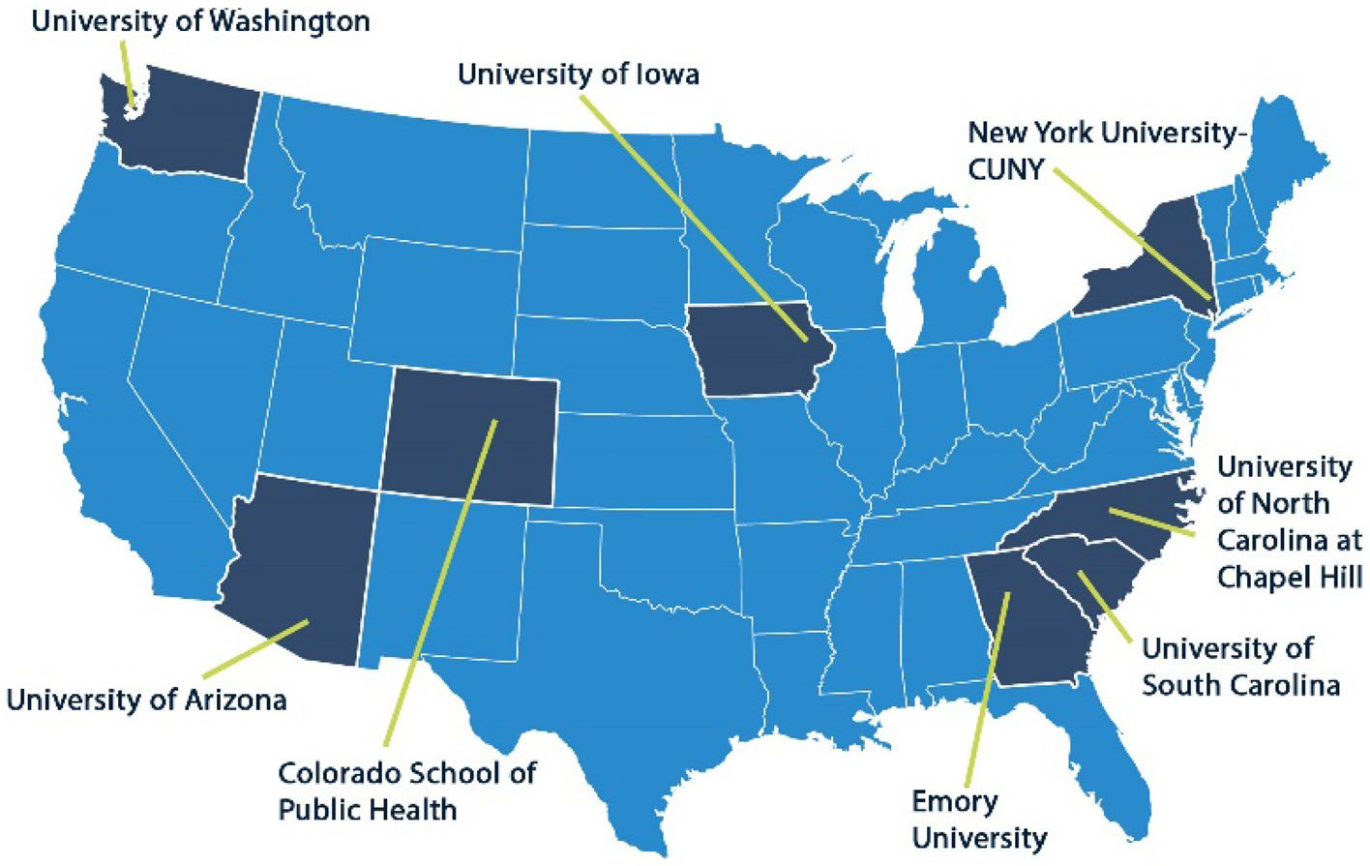
The Eight CPCRN Centers Across the United States

The CPCRN Scholars Cross-Center Workgroup (WG) was formed in 2019 to develop and deliver a D&I Scholars training program, leveraging the resources, infrastructure and expertise of the CPCRN. The WG first delivered the program to 20 scholars in 2021. To evaluate the inaugural program, we conducted a sequential (quantitative + qualitative), mixed methods study [11]. All activities were coordinated by a CPCRN Scholars WG and led by two CPCRN principal investigators and program directors (University of South Carolina and Emory University) and included members of the eight CPCRN centers. The locations of the 8 CPCRN centers are shown on the map in Fig. 1**.** The University of South Carolina IRB approved the evaluation study.

We conducted a baseline survey with all scholars after they volunteered to participate in the evaluation and prior to initiating program training activities in January 2021. At the conclusion of the program in Fall 2021, all scholars were invited to complete a follow-up survey [Supplemental Fig. 1]. We then invited all Scholars and ten of their mentors to participate in 30-min interviews from September to November 2021. Four interviewers completed the interview after verbal consent was given.

The survey included demographic questions and information about their affiliation either their current educational program or organization, in addition to assessing the baseline metrics of D&I competencies. The exit survey asked about overall program evaluation, and attitudes about the program. Scholars were asked to assess their D&I science competencies on a scale of 1-beginner to 3-advanced, as they did at baseline [12]. The 48 competencies encompassed 4 broad domains: (1) definition, background and rationale, (2) theory and approaches, (3) design and analysis, and (4) practice-based considerations. Scholars also rated their satisfaction with each program component, mentoring and the overall program. The final items included questions about use of the Scholar’s program materials in the future, applying D&I science concepts in research and practice, collaborating with fellow CPCRN scholars, and recommendation of the program to others.

An evaluation subgroup of the Scholar’s Workgroup designed the semi-structured guide. A program director administered the survey and sent reminders. The sample included all of the scholars and pre-selected mentors (*n* = 10) to facilitate rapid data collection once the program was complete. The Scholars Program leadership team compiled a list of 15 actively engaged Scholar program mentors whom represented diverse D&I science and geographic regions to participate in the interview. Mentors with availability were interviewed. The exact number of mentors engaged with scholars across the program is unknown due to scholars changing mentors throughout the program and the practice of garnering informal mentorship through workgroup projects. The interview guide included 14 items that covered their overall program experience, reasons for joining the program, description of their project and working with their mentor(s), opinions of the training and webinars, and how the program enhanced their cancer control research, and/or understanding of D&I science. Finally, the guide asked about benefits of the program, suggestions for improvements, and how the program helped them with their career goals. The interview guide for the mentors was composed of 19 questions that covered three domains: mentor experience, Scholar’s project, and the program in general.

The surveys were programmed into Qualtrics [13]. Data were downloaded into SPSS version 26 for analysis [14], and merged for the scholars’ baseline and follow-up data. Descriptive statistics including frequencies and means were run and scales were computed. Data were stratified into two groups (students and practitioner/researchers) in the analyses. For the qualitative analyses, a cancer center’s shared resource conducted the analyses using analyses methods such as reiterative codebook refinement and dual coders [15]. A codebook was developed based deductively on the interview guide; however, other inductive codes were created based on the interview transcript discussions [16]. Two staff independently coded the data based on codebook definitions and met about any discrepancies to reach consensus, shared the coding with the program leads and then conducted thematic analyses of the themes using NVivo 12.0 [17]. Data matrices of the codes were created for the scholars and mentors, and representative quotes were selected by program leads.

## Results

### Baseline and post-program survey findings

All scholars (*n* = 20) completed the baseline survey and 17 completed the exit survey. For the interviews, 18 scholars (90% response rate) and 10 mentors (50%; percentage based on every scholar having one mentor; total number of mentors is unknown) completed the interviews. For the inaugural year, 50% of trainees were students, 45% researchers, and 5% practitioner scholars (Table 1). Scholars overall were 83.3% female and 66.7% White, 27.8% Asian, and 5.5% Hispanic. Nine scholars were students who were at the doctorate level (50%) and from fields such as decision science and health outcomes, epidemiology, and health promotion.

**Table 1:** Demographics of the Scholars (*N* = 18)

Variable	Pre-program survey *N* (%)
Type, *N* = 20	
Student	10 (50%)
Researcher Practitioner	9 (45%) 1 (5%)
Gender	
Female Male	15 (83.3%) 3 (16.7%)
Race	
Asian White Mexican American	5 (27.8%) 12 (66.7%) 1 (5.**5**%)
Student, *N* (%)	10 (55.6%)
In what type of degree granting program are you currently enrolled?	
PhD MSPH	9 (50%) 1 (5.6%)
Year of the program	
First Second Third Fourth	3 (16.8%) 1 (5.6%) 4 (22.3%) 2 (11.2%)
Primary program of study/program concentration	
Clinical Health Psychology Decision Sciences and Outcomes Research Epidemiology Exercise Physiology Health Promotion, Education, and Behavior Organization and Implementation Science Nursing and Health Care	1 (5.6%) 2 (11.1%) 2 (11.1%) 1 (5.6%) 2 (11.1%) 1 (5.6%) 1 (5.6%)

*Competencies* Related to the D&I competencies, scholars’ ratings of their competencies all increased from baseline to posttest (Table 2). Some of the competencies with the greatest change included identifying and applying techniques for stakeholder analysis/engagement (*delta, d* = 0.65), describing relationships between organizational dimensions and D&I research (*d* = 0.54), identifying a process for adapting an evidence-based intervention (*d* = 0.53), and using evidence to evaluate and adapt D&I strategies for specific populations, context, etc. (*d* = 0.48) Although there was not a significant increase in the total D&I competency score (M = 4.39 vs M = 4.56), the scholars reported significant increases from baseline to post-program on all of the subdomains (data not shown). The subdomains that reported the largest increases from the baseline survey were practice-based considerations and D&I design and analyses.

**Table 2:** Rating of Dissemination and Implementation Science Competencies by Scholars’ Group

Variable	Pre-program survey Mean	Post-program survey Mean	Mean Difference
Overall level of experience with dissemination and implementation science?	4.38 (n = 18)	4.55 (n = 18)	0.17
Section A: Definition, Background, and Rationale	(n = 17)	(n = 18)	
A1. Define and communicate D&I research terminology	1.70	1.94*	0.24
A2. Define what is and what is not D&I research	1.76	2.00*	0.24
A3. Differentiate between D&I research and other related areas, such as efficacy research and effectiveness research	1.58	1.88	0.30
A4. Identify the potential impact of disseminating, implementing, and sustaining effective interventions	1.82	1.83	0.01
A5. Describe the range of expertise needed to conduct D&I research	1.52	1.66	0.14
A6. Determine which evidence-based interventions are worth disseminating and implementing	1.76	2.05	0.29
A7. Assess, describe, and quantify (where possible) the context for effective D&I	1.52	1.94*	0.42
A8. Identify existing gaps in D&I research	1.47	1.72	0.25
A9. Identify the potential impact of scaling down (aka de-implementing) an ineffective but often used intervention	1.29	1.77*	0.48
A10. Formulate methods to address barriers of D&I research	1.35	1.72*	0.37
Section B: Theory and Approaches	(n = 17)	(n = 18)	
B1. Describe a range of D&I strategies, models, and frameworks	1.47	1.83*	0.36
B2. Identify appropriate conceptual models, frameworks, or program logic for D&I change	1.47	1.77	0.30
B3. Identify core elements (effective ingredients) of effective interventions, and recognize risks of making modifications to these	1.41	1.61	0.20
B4. Describe a process for designing for dissemination (planning for adoption, implementation, and sustainability during the intervention development stage)	1.41	1.83*	0.42
B5. Describe the relationships between various organizational dimensions (e.g., climate, culture) and D&I research	1.29	1.83*	0.54
B6. Explain how knowledge from disciplines outside of health (e.g., business, marketing, and engineering) can help inform further transdisciplinary efforts in D&I research	1.41	1.66	0.25
B7. Identify and articulate the interplay between policy and organizational processes in D&I	1.23	1.55*	0.32
Section C: Design & Analysis	(n = 17)	(n = 18)	
C1. Describe the core components of external validity and their relevance to D&I research	1.58	1.94	0.36
C2. Identify common D&I measures and analytic strategies relevant for your research question(s)	1.47	1.83	0.36
C3. Identify and measure outcomes that matter to stakeholders, adopters, and implementers	1.64	1.94	0.30
C4. Describe the application and integration of mixed-method (quantitative and qualitative) approaches in D&I research	1.64	2	0.36
C5. Apply common D&I measures and analytic strategies relevant for your research question(s) within your model/framework	1.29	1.77*	0.48
C6. Identify possible methods to address external validity in study design reporting and implementation	1.41	1.61	0.20
C7. List the potential roles of mediators and moderators in a D&I study	1.29	1.83*	0.54
C8. Identify and articulate the trade-offs between a variety of different study designs for D&I research	1.41	1.66	0.25
C9. Describe how to frame and analyze the context of D&I as a complex system with interacting parts	1.29	1.83*	0.54
C10. Effectively integrate the concepts of sustainability/sustainment and the rationale behind them in D&I study design	1.29	1.55	0.26
C11. Describe gaps in D&I measurement and critically evaluate how to fill them	1.29	1.66*	0.37
C12. Effectively explain and incorporate concepts of de-adoption and de-implementation into D&I study design	1.11	1.55*	0.44
C13. Incorporate methods of economic evaluation (e.g., implementation costs, cost-effectiveness) in D&I study design	1.29	1.55*	0.26
C14. Evaluate and refine innovative scale-up and spread methods (e.g., technical assistance, interactive systems, novel incentives, and “pull” strategies)	1.11	1.44*	0.33
Section D: Practice-Based Considerations	(n= 17)	(n = 18)	
D1. Describe the importance of incorporating the perspectives of different stakeholder groups	2	2.38*	0.38
D2. Describe the concept and measurement of fidelity	1.76	1.94	0.18
D3. Articulate the strengths and weaknesses of participatory research in D&I research	1.58	1.94*	0.36
D4. Determine when engagement in participatory research is appropriate with D&I research	1.41	1.94*	0.53
D5. Describe the appropriate process for eliciting input from community-based practitioners for adapting an intervention	1.58	1.83	0.25
D6. Identify and apply techniques for stakeholder analysis and engagement when implementing evidence-based practices	1.29	1.94*	0.65
D7. Identify a process for adapting an intervention and how the process is relevant to D&I research	1.41	1.94*	0.53
D8. Explain how to maintain fidelity of original interventions during the adaption process	1.17	1.94*	0.77
D9. Identify sites to participate in D&I studies, and negotiate or provide incentives to secure their involvement	1.52	1.55	0.03
D10. Identify and develop sustainable partnerships for D&I research	1.47	1.61	0.14
D11. Describe how to measure successful partnerships for D&I research	1.23	1.61*	0.38
D12. Use evidence to evaluate and adapt D&I strategies for specific populations, settings, contexts, resources, and/or capacities	1.29	1.77*	0.48

* *p* < 0.5 for means test

*Engagement* Related to the curriculum, 94.4% reported attending the *PPHEIA* webinars and 77.8% reported completing the NCI Dissemination and Implementation modules (Table 3). About 33% completed their project, while 55.6% partially completed it. A large majority (94%) reported receipt of mentoring or partial mentoring in the program. About 78% of scholars engaged with a CPCRN workgroup. The program components that were ranked most valuable (defined as very or moderately valuable) were the PPHEIA training (88.9%), NCI D&I training modules (83.3%), and webinars (kickoff webinar-88.9% and selecting implementation theories and models-88.9%). Two other components were rated moderately valuable: mentoring (72.2%) and engagement with CPCRN project or workgroup (72.2%).

**Table 3:** Overall Scholars Program Evaluation (*N*− = 18)

	Post-program evaluation^a^, N (%)		
	Researchers/ practitioners *n* (%)	Students *n* (%)	Total *N* (%)
Ability to complete your project			
Yes Partially Not Applicable	1 (12.5) 6 (75.0) 1 (12.5)	3 (30.0) 5 (50.0) 2 (20.0)	4 (22.2%) 11 (61.1%) 3 (16.7%)
Engagement with a CPCRN workgroup			
Yes No	4 (50.0) 4 (50.0)	10 (100) –	14 (77.8%) 4 (22.2%)
Completion of Scholar’s Program Components			
Putting Public Health Into Action—Educational Training			
Yes	7 (100)	10 (100)	17 (94.4%)
NCI Dissemination and Implementation Modules			
Yes Partially	8 (100) –	6 (75.0) 2 (25.0)	14 (77.8%) 2 (11.1%)
Implementation in Action: CPCRN Investigators’ Experiences—March 31st Webinar			
Yes Partially	6 (85.7) 1 (14.3)	7 (77.8) 2 (22.2)	13 (72.2%) 3 (16.7%)
Practitioner—August 5th Webinar			
Yes Partially	5 (100) –	5 (83.3) 1 (16.7)	10 (55.6%) 1 (5.4%)
Individual project			
Yes Partially	2 (28.6) 5 (71.4)	4 (44.4) 5 (55.6)	6 (33.3%) 10 (55.6%)
Received mentoring within the program			
Yes Partially	3 (37.5) 5 (62.5)	5 (55.6) 4 (44.4)	8 (44.4%) 9 (50%)
Engagement with CPCRN project or work group(s)			
Yes Partially	4 (80.0) 1 (20.0)	8 (80.0) 2 (20.0)	12 (66.6%) 3 (16.7%)
Valuable Components of the CPCRN Scholars Program			
Putting Public Health Into Action—Educational Training			
Very/Moderately Valuable Somewhat Valuable A Little Valuable	7 (100) – –	9 (90.0) 1 (10.0) –	16 (88.8%) 1 (5.6%) –
NCI Dissemination and Implementation Modules			
Very/Moderately Valuable Somewhat Valuable A Little Valuable	7 (87.5) 1 (12.5) –	8 (100) –	15 (83.3%) 1 (5.6%) –
Kick-Off—Jan. 25th Webinar			
Very/Moderately Valuable Somewhat Valuable A Little Valuable	7 (87.5) 1 (12.5)	9 (90.0) – 1 (10.0)	16 (88.9%) **-** 2 (11.2%)
Implementation in Action: CPCRN Investigators’ Experiences—March 31st Webinar			
Very/Moderately Valuable Somewhat Valuable A Little Valuable	7 (87.5) 1 (12.5)	7 (77.8) 2 (22.2) –	14 (77.7%) 2 (11.1%) 1 (5.6%)
Selecting and Applying Implementation Theories, Models and Frameworks—June 7th Webinar			
Very/Moderately Valuable Somewhat Valuable A Little Valuable	7 (87.5) – 1 (12.5)	9 (90.0) – 1 (10.0)	16 (88.9%) – 2 (11.1%)
Practitioner—August 5th Webinar			
Very/Moderately Valuable Somewhat Valuable A Little Valuable	6 (100) – –	6 (85.8) 1 (14.3) –	12 (66.6%) 1 (5.4%) –
Wrap-Up			
Very/Moderately Valuable Somewhat Valuable A Little Valuable	3 (50.0) 2 (33.3) 1 (16.7)	9 (100) – –	12 (66.6%) 2 (11.1%) 1 (5.6%)
Individual project			
Very/Moderately Valuable Somewhat Valuable A Little Valuable	5 (71.4) 1 (14.3) 1 (14.3)	7 (87.5) 1 (12.5) –	12 (66.6%) 2 (11.1%) 1 (5.6%)
Mentoring within the program Very/Moderately Valuable Somewhat Valuable A Little Valuable	5 (62.5) 3 (37.5) –	8 (88.9) 1 (11.1) –	13 (72.2%) 4 (22.2%) –
Engagement with CPCRN project or work group(s)			
Very/Moderately Valuable Somewhat Valuable A Little Valuable	3 (50.0) 3 (50.0) –	10 (100) – –	13 (72.2%) 3 (16.7%) –

^a^The number of participants varied by item. Responses for the total were out of 18 and there are some responses missing in some cases due to some unanswered questions by scholars. Therefore, the total sometimes does not add to 100%

*Satisfaction* For overall ratings of the program, 77.8% reported satisfaction with the format of the activities and ability to speak of D&I concepts (Table 4). Overwhelmingly, trainees agreed that knowledge about the CPCRN increased (88.9%). About 83% reported being very or somewhat satisfied with the mentoring received, 72.2% reported strongly agreed or agreed that their mentor helped engage them with a workgroup, and 66.6% reported that their mentor expanded their network. All scholars (100%) reported that they would use the curriculum materials in the future and would recommend the program to others.

**Table 4:** Satisfaction with Program and Learning Transfer (*N* = 18)

Satisfaction with the level of mentoring that you received^a^ Neutral Somewhat Satisfied Very Satisfied	1 (12.5) 4 (50.0) 3 (37.5)	2 (20.0) 4 (40.0) 4 (40.0)	3 (16.7%) 8 (44.4%) 7 (38.9%)
Satisfaction with the educational opportunities and resources provided			
Strongly Disagree/Disagree Neutral Agree/Strongly Agree	– 1 (12.5) 7 (87.5)	1 (10.0) 1 (10.0) 8 (80.0)	1 (5.6%) 2 (11.1%) 15 (83.3%)
Overall, I was satisfied with the webinar presentations			
Strongly Disagree/Disagree Neutral Agree/Strongly Agree	– – 8 (100)	– – 10 (100)	– – 18 (100%)
Overall, this training was very relevant to my work or academic studies	–		
Strongly Disagree/ Disagree Neutral Agree/Strongly Agree	1 (12.5) 7 (87.5)	– – 10 (100)	– 1 (5.6%) 17 (94.4%)
I was satisfied with the format of the activities			
Strongly Disagree/ Disagree Neutral Agree/Strongly Agree	– 2 (25.0) 6 (75.0)	1 (10.0) 1 (10.0) 8 (80.0)	1 (5.6%) 3 (16.7%) 14 (77.8%)
I was satisfied with the timing and frequency of activities for this training program			
Strongly Disagree/ Disagree Neutral Agree/Strongly Agree	– 1 (12.5) 7 (87.5)	3 (30.0) 1 (10.0) 6 (60.0)	3 (16.7%) 2 (11.1%) 12 (72.2%)
I am able to speak confidently about D&I practices Strongly Disagree/Disagree Neutral Agree/Strongly Agree	– 1 (12.5) 7 (87.5)	1 (11.1) 2 (22.2) 7 (77.7)	1 (5.6%) 3 (16.7%) 14 (77.8)
My mentor was very helpful in expanding my network			
Strongly Disagree/Disagree Neutral Agree/Strongly Agree	1 (12.5) 1 (12.5) 6 (75.0)	1 (10.0) 3 (30.0) 6 (60.0)	2 (11.1%) 4 (22.2%) 12 (66.6%)
My CPCRN research mentor was instrumental in helping me engage within the CPCRN workgroup Strongly Disagree/Disagree Neutral Agree/Strongly Agree	– 2 (25.0) 6 (75.0)	1 (10.0) 2 (20.0) 7 (70.0)	1 (5.6%) 4 (22.2%) 13 (72.2%)
I learned a lot about CPCRN Strongly Disagree/Disagree Neutral Agree/Strongly Agree	– 1 (12.5) 7 (87.5)	– 1 (10.0) 9 (90.0)	– 2 (11.1%) 16 (88.9%)
Overall, I felt it was valuable to learn about the CPCRN Agree/Strongly Agree	8 (100)	10 (100)	18 (100)
I felt prepared to be participating in a CPCRN workgroup because of the CPCRN Scholars program Strongly Disagree/Disagree Neutral Agree/Strongly Agree	1 (12.5) 1 (12.5) 6 (75.0)	2 (20.0) 1 (10.0) 7 (70.0)	3 (16.7%) 2 (11.1%) 13 (72.2%)
Use the CPCRN Scholars Program materials in the future? Yes	8 (100)	10 (100)	18 (100%)
Apply any D&I science concepts or practices to any of your future papers or projects? Yes	8 (100)	10 (100)	18 (100%)
Keep a working relationship with your mentor(s)? Extremely/Somewhat Likely Neither Likely nor Unlikely A Little Likely	7 (87.5) – 1 (12.5)	10 (100) – –	17 (94.4%) – 1 (5.6%)
Keep a working relationship with your fellow CPCRN Scholar peers? Extremely/Somewhat Likely Neither Likely nor Unlikely A Little Likely	5 (62.5) 3 (37.5) –	7 (70.0) 1 (10.0) 2 (20.0)	12 (66.6%) 4 (22.2%) 2 (11.1%)
Recommend this program to others Extremely/Somewhat Likely	8 (80.0)	10 (100)	18 (100%)

^a^The number of participants varied by item. Responses for the total were out of 18 and there are some responses missing in some cases due to some unanswered questions by scholars. Therefore, the total sometimes does not add to 100%

### Post-program interviews with scholars

Scholars reported a variety of benefits of the program. Scholars reported that learning implementation strategies, frameworks, and methods was useful (*n* = 6). One scholar stated, *“So it's introduction to implementation science, theory, methods, and frameworks, implementation strategies. So these are also very useful. But it's always more interesting at the—at least for me it's very interesting to hear from people actually doing projects on the ground because it helps you.”* Some scholars (*n *= 4) noted that the program’s variety of teaching methods and mediums such as using websites, PowerPoints, modules, and a variety of other tools, were very beneficial aspects of the program. Some participants (*n* = 3) found that the curriculum helped with their work outside of the program as a teaching assistant, or through a class assignment, dissertation, or another project.

Scholars were asked to share their experiences in the CPCRN Scholars program. Several scholars (*n* = 4) shared that due to the CPCRN program they have more knowledge about D&I research: “*I think one of the things that I got the most out of it was just the educational experience, getting a better grasp and understanding of D&I research and supporting building a foundation to it. That’s probably what I got the most out of it, was just through the webinars and, in the readings, became much more, I guess, knowledgeable about D&I research. That’s probably what I benefitted or enjoyed the most out of it*.” Participants shared how the program was beneficial for their careers (*n* = 4), for their own research (*n* = 2), and for building their network (*n* = 2). Regarding how the program benefited one’s career, one scholar shared: “*I think it was a nice addition to what my work has been as a project director. I feel like there was a fair amount of overlap, and it allowed me to really be integrated into the network, and it also allowed me to have a better understanding with D&I science and its scope and its range since that's not my area of study currently*…”.

We assessed the scholars’ perspectives on the workgroup opportunities in the program and their involvement in workgroups. Scholars reported a positive aspect of the workgroups was the opportunity the workgroup provided to meet others, network with people, and collaborate with other scholars on projects (*n* = 3). For example, one scholar described how she was able to collaborate with another scholar from a different institution on a K award (an award funded by the National Institutes of Health (NIH) to provide support to junior faculty on their research) due to shared research interests and both their expertise complemented each other. A few scholars described workgroups as inviting and reported workgroups had an inclusive, positive culture (*n* = 2). Some workgroups met more frequently than others. Scholars involved with workgroups who met frequently found that helpful. For example, one scholar stated: *“There is a biweekly meeting that we had with a smaller health equity group just to keep the project going. So that was very helpful.”* In workgroups, scholars were able to get feedback from collaborators outside of their institution (*n* = 2) and they gained an understanding of the structure and inner workings of the workgroup and its projects (*n* = 2). One scholar stated: *“And people would seek broader opinion from the group, and that is how I had an experience getting feedback from the other collaborators who are not associated with the institution. So this is again a great learning experience, especially at a student level. So it is my first time working with so many collaborators from different universities.”* One major disadvantage that participants (*n* = 5) noted was not joining a workgroup during their time in the program, and other challenges to workgroup engagement included having trouble joining a workgroup (*n* = 1), workgroups conflicting with their class schedule (*n* = 1), and workgroups having infrequent meetings (*n* = 1). For example, one scholar stated: “*I tried the organizational readiness one. But I think only one of the meetings actually happened. The rest of them were canceled. And the one that happened I was in an all-day board meeting. So I couldn’t attend.*”

Other valuable program components were mentioned. One positive effect of the program on the scholars' networks was that it provided access to future collaborators on papers and post-doctoral positions (*n* = 3). One participant stated: “*I have made connections with a couple of faculties to get into my PhD research work. So yes, I have made connections, and it was very helpful networking with people through my participation as a scholar in this program*.” Select scholars (*n* = 3) mentioned that the program helped them expand connections through access to the established CPCRN network or professionals. Other ways the CPCRN program impacted the scholars’ networks included facilitating connections with different academic centers (*n* = 2), the ability to make connections through the workgroups (*n *= 2), and getting to know people outside of the scholar’s home institution (*n* = 2). Table 5 presents a joint display of some evaluation results with the program’s quantitative ratings and exemplar quotes from different types of scholars.

**Table 5:** Joint Analyses of Survey and Interviews of CPCRN Scholars

Scholars Program Component	Measures/Item from Survey and Interviews	Score [QUANT]	Qualitative Interview Quotes [QUAL]
Project	Survey Value of Individual Project	66.7% Very/Moderately valuate	*I participated in the rural cancer work group, and was then made a part of the collaborative work. And they assigned me a project on analyzing like or navigating data—existing secondary data resources to address rural cancer control. And we formulated some information, and put them all together in the form of a tip sheet. And it is still in progress. And we are collaborating with the American Cancer Society for their input… [Student]* *And I reached out to the folks that were on that project, as I've been involved in a number of different systematic reviews, so I knew I could contribute in a meaningful way and kind of had the skills, and that it was a project I was interested in, because I am particularly interested in childhood and adolescent and young adult survivors, so kind of the pediatric cancer population appealed to me. So, yeah, I joined in on that early, kind of helped with article selection. So the search strategy was done, and then me and, it was a research assistant of someone in the survivorship work group, kind of working on article selection and then kind of moving through that process of selection and data abstraction, and we are now in the phases of putting manuscript tables together [Student]* *So this group was formed to compile a set of guiding health and racial equity principles to help orient the work of CPCRN centers. And also come up with operational definitions and measurement tools to monitor and track progress on these indicators. So my role as a CPCRN Scholar was to help support this project* *And so what I've done is helped lead a—like a scan of frameworks, social determinants of health, health equity frameworks used by the CPCRN Centers. And then we did a center-wide survey, a network-wide survey sorry, to under – to try to understand which frameworks are being used. Oh, I apologize, after we reviewed the frameworks that the centers used we came up with a list of—a draft list of guiding principles. [Post doc scholar]* *So for my project, I worked on a—I did qualitative interviews on facilitators and barriers for survivorship care, those who survived pediatric cancer, and I'm still working on the project, I haven't finished, but just analyzed, looking for themes in the data and then putting it into a fishbone diagram. [Practitioner]*
Curriculum Putting Public Health Evidence into Action NCI Dissemination and Implementation Modules	Survey Value of PPHEIA Value of NCI D&I Modules	88.9% Very/Moderately valuate 88.3%	*That training really laid out steps to getting to implementation science and one of the projects we’re working on is kind of one of those earlier stages and we’re trying to write a manuscript and think about the next steps. And I think the training really helped me establish a knowledge base of – we’re starting on this step. Here’s what we can do next and here’s resources involved with that* *Secondly, the training module was very beneficial. The resources that were provided through those trainings, I think those were great. Thirdly, the whole program really helped me in adding an aspect of implementation science to my dissertation. [Student]*
Mentoring	Survey & Interview Value of mentoring within the Program	72.2% Very/Moderately valuate	*Yeah, so Dr. T here—I mean she’s my life-long mentor, to be honest. She’s my key mentor now—that relationship is wonderful. Yeah, Dr. W kind of just adopted her as an informal mentor throughout the process, just getting guidance when needed. Again, I think the mentorship process—the mentors are very committed, and that’s pretty clear. C as well, right? Everybody would show up and, again, the energy. So I will say that overall, the mentorship team of the scholars was phenomenal, a very dedicated group, and that showed through I think for everybody*
Webinar	Survey & Interview Satisfaction with webinars	100% Strongly agreed or Agreed	*Informative Webinars* *I watched a few of those webinars and then I did participate on some of the live ones where you had to be at the Zoom meeting type of thing. And both of those were good, one was about, I think, about practice and providers…But yeah they were all informative just because I have not done implementation science before and a variety of things* *I remember it was I think C who presented on her project. And it's just seeing how –. But then when you see them implemented in an actual project you figure out what they mean. And how they're actually operationalized in practice, so that was very, very useful. And I would like more of this. Because that's basically how you learn. You can read about it for sure, and it gives you some background information. But then when you talk to someone who has actually done the work it helps to see what are the challenges of this? What are the benefits of this? Would I consider it in a project going forward? So that was extremely helpful* *Helpful Recordings* *There is a good amount of webinars. They weren't too much, so it was—I think it was easy to commit to going to all of them that were offered. And then they even have their recording option available like if you weren't able to attend*
Workgroup	Survey & Interview Value of engagement in Workgroup(s)	72.2% Very/Moderately valuate	*And also another benefit is that through the workgroups because in the beginning, we had the meeting with all the scholars, and we shared our research interest, and I was able to connect with Dr. [X]. So he’s from University of Arizona. So after the first introduction meeting, we met separately, and we found that for his current K award, there are come components we can actually collaborate together, so our expertise will be really complementary to each other* *And so uh, through our participation it is like each and every goals of the project would be discussed along with the timeline and their current progress on them. And people would seek broader opinion from the group, and that is how I had an experience getting feedback from the other collaborators who are not associated with the institution. So this is again a great learning experience, especially at a student level*
CPCRN network	Survey & Interview Learned a lot about CPCRN	88.9% Strongly agreed or Agreed	*You know, I have research interests in this area, and kind of having that connection to this really expansive network of folks across the country, I think, is kind of the most meaningful for me moving forward and kind of seeing the different types of research that are happening across the network, and just kind of having that established relationship that I was part of this program, and moving forwards, thinking about collaborations on future papers, looking for postdocs, *etc *I have made connections with a couple of faculties to get into my PhD research work. So yes, I have made connections, and it was very helpful networking with people through my participation as a scholar in this program*
Overall program	Survey & Interview Overall satisfaction with the program and resources	88.3% Strongly agreed or Agreed	*I think one of the things that I got the most out of it was just the educational experience, getting a better grasp and understanding of D&I research and supporting building a foundation to it. That’s probably what I got the most out of it, was just through the webinars and, in the readings, became much more, I guess, knowledgeable about D&I research. That’s probably what I benefitted or enjoyed the most out of it* *But yeah—every webinar or any conversation I attended was very helpful. So either it provided some information on D&I or just in general cancer research or projects that I'm like, “Uh, that’s a really interesting approach, that’s something I can bring to my work or something else.” So basically everything I attended yeah was—when I could, it was worth my time which I really much appreciated* *So overall, really enjoyed it and found it very valuable for kind of where I am right now, in terms of looking forward to a career and kind of developing my own research interests and thinking about kind of long-term research goals and career goals*

### Post-program interviews with mentors

Mentors were asked questions about the CPCRN Scholars program, including their general impressions, challenges, purpose of the program, and the most important area of the program. Mentors shared that interactions with scholars happened during individual meetings generally consisting of discussion around the scholar’s project and training activities. Interactions were reported to be smooth and easy, with some discussions broadening beyond the CPCRN scholar training. Three mentors stated that they interacted with scholars at scheduled meetings, with one participant stating, “*I think we built it into our weekly or bi-weekly meetings so she would let me know how many interviews she’s done, where she is on analysis*.”

Mentors shared their thoughts about the most important aspects of the Scholars’ program. Three mentors mentioned this was networking, with one participant saying*, “…the most impactful was getting people integrated into this larger network. And realizing—allowing them to realize the richness of experts across the nation who are working in D&I science that they can link into. I think that was the most impactful.”* Four mentors discussed their general satisfaction with the program, with one saying “*I think it has been a great program and a real benefit to the participants…*.” Three mentors mentioned that the program allows scholars to build relationships, with one explaining the importance of keeping scholars engaged; one commented, “*I think having the program is a good opportunity to get people engaged in the work of the CPCRN. I liked that, at least it seemed like, from a worker perspective, you were trying to match scholars with these different workgroups to collaborate with other investigators. I think building those networks and relationships is really important to do. The sooner you can do them, the better. Yes, I think pretty favorably of the program*.” Table 6 presents key findings related to mentors’ perceptions of the program.

**Table 6:** Interviews with Mentors in the Program

Scholars Program Component	Measures/Item from Interviews	Qualitative Interview Quotes [QUAL]
Importance of CPCRN Scholars Program	Value of the CPCRN Scholars Program	*I don't know if I'd say the word critical, but the most impactful was getting people integrated into this larger network. And realizing—allowing them to realize the richness of experts across the nation who are working in DNI science that they can link into. I think that was the most impactful.* [Researcher and Doctoral Mentor]
Impressions of the Scholars Program	General impressions	*And then of course our updated CPCRN Putting Public Health Evidence Into Action. We always update that. So they’re getting the most contemporary thinking and skills. And then the other thing that I think benefitted her was she found—if you want to call it an affinity group because we’re doing survivorship work—and she met other practitioners and researchers in the survivorship work group.* [Practitioner and Post-doctoral Mentor]
Mentor–Mentee Relationship	Value of mentoring within the Program	*Not formally for that project since we’d already sort of since she was working with me as a GA that we kind of pulled it into that. So I guess not in a formal—anything formally for the scholar’s program, but just kind of pulled it under the other work that she was doing.* [Graduate Student Mentor]
Mentor Meaning	Enjoyment of mentorship	*Just being very available for the mentees to be able to come to them if they have any questions, I think being a guide to help them think through potential research interests or areas and guiding them through that process, just depending upon their level of knowledge or information on how to do that. I’m trying to think in terms of other qualities. Yes, I think just setting up regular expectations and setting regular meetings, being available when possible, and working to create those roles and responsibilities collectively; and just serving as a support system. I think most of the scholars, if I’m not mistaken, are post-doc level, right?* [Doctoral Student Mentor]
Mentor Support	Involvement in mentee’s project	*And so, creating that tip sheet is something she’s working on. So for me, what I’m doing is providing feedback as she drafted it, providing guidance or making suggestions on different elements to put in there, connecting her with other people who have been involved—like the previous co-chair of the rural cancer work group and then one of the work group members who’s done some similar kinds of documents who works at the American Cancer Society. So that’s kind of been my role. Just to provide feedback or guidance or connections for our specific project.* [Graduate Student Mentor]
	Direct assistance in mentee’s project	*So yeah, I developed that. But she actually did the data collection and did the analysis. But I also reviewed her analysis, her thematic analysis, because again the meta fishbone had all 11 sites and we actually counted the themes that came up. I kind of reviewed those themes and also made suggestions for—you know, like if she grouped themes under a thematic topical area to make sure that I understood it so that if she was presenting like at the D&I conference and also with an advisory meeting tomorrow to other oncologists and people who work in the pediatric space, that they would understand.* [Practitioner and post-doc Mentor]
Scholars’ Program Expectations	Expectations Unclear	*Yes, I think in the [Name of workgroup], again, in my mind I wasn’t clear on exactly the expectations of what was involved in with the scholars, and I didn’t get anyone that said, “here’s exactly what I need to be doing, and here’s what the workgroup can do for me.” I think I had some of those conversations with [mentee], but not much. I would say my involvement with the scholars within the context of the workgroup would just be like with any other member, setting the stage in terms of the roles and responsibilities, of what the activities we’re going to be doing are. [Doctoral Student and post-doctoral/ workgroup Mentor]*
Experience	Negative Comments/Experience	*Yeah, so [Name of Scholar]—and [Name of Scholar] was in the process of—he was in the job market and so his—our time together in the scholar’s program was probably dominated by not – you know, mentorship it’s not like I was—I’d take off my post-doc mentor hat and then put on my CPCRN scholar’s program hat. It was like all together. But we never explicitly focused or discussed the CPCRN scholar’s program. Same with [Name of another Scholar]. I don’t think I had any one-on-ones with her. As a work group leader, my effort for her was just to engage her to the extent that she wanted to be and be responsive when she expressed interest in participating in different things. But suffice it to say, all very unstructured and limited. [Post-doc Mentor]* *And the other thing that I feel like I didn’t do as well is that I should have attended the scholar work group meetings and I just had so many other commitments, I could not. And maybe I would have had better direction for—because I sort of felt worthless at times. Like I don’t know where I should be taking them. [Practitioner and Student Mentor]*

This mixed methods evaluation also asked about recommendations for program improvements. Open-ended questions on the survey and an interview item were included to allow respondents to suggest programmatic changes. Recommended areas for improvements from scholars included greater opportunities for networking and mentoring, more communications around program expectations, and extending the length of the program or project completion timeline. For example, many students (*n* = 5) shared that there was little to no interaction with other scholars. One participant specifically shared, “*I had a chance to interact with them {fellow CPCRN Scholars}, but not much. Just it would have been an introductory interaction kind of thing. But I was not able to continue interacting with them*.” Similarly, mentors wanted more information about the program and expectations to help them with their roles.

## Discussion

Overall, we found high ratings for the inaugural year of the Scholars Program across many program components. Scholars valued the curriculum, engagement with an established, national cancer research network and advising by their mentors. Generally, the scholars rated the two D&I curricula and engagement with the CPCRN higher than other program components. Their learning was evident in changes in D&I competencies across many topical domains. Related to mentoring, scholars who already had a strong working relationship with a current mentor or their mentor was already active in the CPCRN reported having the following: more engagement with their mentor, their mentor helped the scholar engage them with a workgroup, linked them to other researchers in their areas of interest, and a positive mentorship experience. Expansion of their network is critical since many of the scholars were students and this may build their relationship to the fields of D&I science and cancer control.

We found that scholars highly rated the fact that the training was in the context of an established national research network: learning about the history of the CPCRN, working within CPCRN topic-focused workgroups, and networking nationally with members located in geographically diverse settings as part of the program. The unique multi-institutional infrastructure of the CPCRN and its many topical workgroups around cancer, health equity, and health-related translational research projects provides many opportunities for the scholars for engagement and real-world application.

In turn, scholars catalyzed efforts and related outputs of the workgroups. For example, several scholars contributed to workgroup projects (e.g., equity checklist, data collection, literature reviews). This participation resulted in submission of abstracts and manuscript writing with a cohort of researchers and practitioners. Scholars who engaged in workgroups that met frequently reported a higher positive experience compared to scholars who were involved with workgroups who met on rare occasions or could not attend the meeting due to other commitments. Additional, scholars improved in their competency around contemporary D&I issues of health equity and community engagement (e.g., program adaptation, partnership engagement); researchers and practitioners have promoted these critical concepts and skills in practice [18–20]. Future evaluations (e.g., a planned scholar alumni survey) can explore the long-term impacts of this engagement for Scholar’s career development and continued engagement with the CPCRN.

The Scholars program fulfills the CPCRN goals to build the D&I workforce. Research has demonstrated the need for more D&I training and building capacity of various professionals across career stages [21, 22]. This program is unique since many D&I training programs are focused on researchers and it expands the trainees to include both students in public health and practitioners. In addition, to address health inequities, D&I scholars should be diverse and come from different setting; this program joins other national efforts to address health equity in translational science [23]. Future research could evaluate the program’s influence on the student scholars’ trajectory and how the program is helping to increase the pipeline of diverse scholars in D&I for cancer prevention and control and other outcomes specified in our program logic model [6].

The evaluation results from the inaugural year of implementation identified several areas for continuous process improvements. Scholars reported a desire for the program to be longer, with more interactions with each other. The flexibility of the program’s structure was greatly appreciated by all, however, explicitly communication about program roles and expectations was requested. For example, some of the tracks had extra meetings for check-in with the scholars while others did not. Expectations for mentor–mentee interactions could be clarified, as those frequently engaging found this valuable. Similarly, engagement in WG was reported to be important to program satisfaction. Importantly, the inaugural training program was launched during the international pandemic of COVID-19, thus limiting opportunities for face to face interactions which may have furthered engagement and possibly improved communications, but did allow for digital and later accessible webinars.

The inaugural year, during the height of the pandemic, served as a soft launch of the program. It was encouraging to see the number of represented institutions grew by seven, including one that is international, Swiss Tropical and Public Health Institute) in its second year (2021–2022 Scholars Cohort). The 2022–2023 Scholars applicant pool indicates continued strong growth with a 135% increase of applications from the second year and the 2022–2023 cohort consists of 21 schools and organizations globally. Programmatically, we had fewer practitioners who applied during the inaugural year of the program than in the second year of the program. Recruitment channels were expanded with partnering agencies and funders to increase awareness of the program among this group. The Scholars Workgroup made program modifications based on the evaluation results in extending the program by a few months (9 to 11), providing more information about the program and its components in advance of enrollment, establishing more frequent communications overall, providing additional information for mentors about the program and mentor expectations, and adding virtual Scholars mixers to the programming. The mixers were added for scholars to voluntarily share a few PowerPoint slides about their project aims and methods, gain input from other scholars and mentors and refine outputs and next steps. These additions were to enhance peer exchange and collaboration which have been identified as capacity-building mechanisms [24].

Strengths of this program evaluation study included use of mixed methods to assess opinions of the program and in-depth feedback about its components qualitatively. The data collection covered a breadth of topical domains for the evaluation from ratings of program components, D&I competencies, mentoring, and career developments. We included the mentors as part of the evaluation. However, the evaluation results may not be generalizable to other D&I trainees of students, researchers, or professionals as our training program has unique features. All data were based on self-report and information on progress on projects were not validated with the mentors. We interviewed a lead mentor for only half of the Scholars due to time and funding constraints. Future evaluations could include perspectives of all mentors.

## Conclusion

The inaugural CPCRN Scholar program year yielded positive results, particularly related engaging with about the CPCRN and increasing knowledge about D&I science and cancer control among its student, researcher, and practitioner scholars. There were many reported benefits, largely centered on new professional relationships, connections and networking, and some suggestions for improvements. This training program can build the capacity of current and future researchers and practitioners in D&I science. Future research could explore the long-term impact of the CPCRN D&I scholar training and networking on career trajectories in cancer prevention and control as well as cancer health disparities research.

## Supplementary Information

Below is the link to the electronic supplementary material.

Supplementary file1 (DOCX 43 KB)

## Data Availability

The datasets generated during and/or analyzed during the current study are not publicly available due to each scholar’s privacy but are available from the corresponding author on reasonable request.
