# Supplementary material for: Mixed methods evaluation of the inaugural year of the Cancer Prevention and Control Research Network’s (CPCRN) scholars program
Source: Cancer Causes Control. 2023 Apr 28;34(Suppl 1):57–73. doi: 10.1007/s10552-023-01702-1 (PMC10139907; doi:10.1007/s10552-023-01702-1)
Supplement: Supplementary file 1 — Supplementary file1 (DOCX 43 KB) [file 10552_2023_1702_MOESM1_ESM.docx]

Supplemental Figure.

Figure 1. CPCRN Scholar Program Timeline
